# CMV Retinitis in the Context of SARS-CoV-2 Infection: A Case Study and Comprehensive Review of Viral Interactions

**DOI:** 10.3390/pathogens13110938

**Published:** 2024-10-29

**Authors:** Emil Robert Stoicescu, Laura Andreea Ghenciu, Roxana Iacob, Adina Iuliana Ardelean, Ecaterina Dăescu, Ovidiu Alin Hațegan, Diana Manolescu, Emanuela Tudorache, Casiana Boru, Mirabela Dima

**Affiliations:** 1 Radiology and Medical Imaging University Clinic, ‘Victor Babes’ University of Medicine and Pharmacy Timisoara, Eftimie Murgu Square No. 2, 300041 Timisoara, Romania; stoicescu.emil@umft.ro (E.R.S.); dmanolescu@umft.ro (D.M.); 2 Research Center for Medical Communication, ‘Victor Babes’ University of Medicine and Pharmacy Timisoara, Eftimie Murgu Square No. 2, 300041 Timisoara, Romania; roxana.iacob@umft.ro; 3 Field of Applied Engineering Sciences, Specialization Statistical Methods and Techniques in Health and Clinical Research, Faculty of Mechanics, ‘Politehnica’ University Timisoara, Mihai Viteazul Boulevard No. 1, 300222 Timisoara, Romania; 4 Department of Functional Sciences, ‘Victor Babes’ University of Medicine and Pharmacy Timisoara, Eftimie Murgu Square No. 2, 300041 Timisoara, Romania; 5 Center for Translational Research and Systems Medicine, ‘Victor Babes’ University of Medicine and Pharmacy Timisoara, Eftimie Murgu Square No. 2, 300041 Timisoara, Romania; 6 Department of Anatomy and Embriology, ‘Victor Babes’ University of Medicine and Pharmacy Timisoara, 300041 Timisoara, Romania; daescu.ecaterina@umft.ro; 7 Discipline of Ophtalmology, ‘Victor Babes’ University of Medicine and Pharmacy Timisoara, 300041 Timisoara, Romania; milcu.adina@umft.ro; 8 Discipline of Anatomy and Embriology, Medicine Faculty, “Vasile Goldis” Western University of Arad, Revolution Boulevard 94, 310025 Arad, Romania; hategan.ovidiu@uvvg.ro (O.A.H.); boru.casiana@uvvg.ro (C.B.); 9 Center for Research and Innovation in Precision Medicine of Respiratory Diseases (CRIPMRD), ‘Victor Babes’ University of Medicine and Pharmacy Timisoara, 300041 Timisoara, Romania; emanuela.tudorache@umft.ro; 10 Department of Neonatology, ‘Victor Babes’ University of Medicine and Pharmacy Timisoara, Eftimie Murgu Square No. 2, 300041 Timisoara, Romania; dima.mirabela@umft.ro

**Keywords:** CMV retinitis, SARS-CoV-2, CMV interaction, COVID-19, cytomegalovirus, AIDS-related CMV retinitis, ocular complications in COVID-19, cytomegalovirus reactivation in SARS-CoV-2, AIDS-related ocular complications, HIV, CMV, SARS-CoV-2 coinfection, valganciclovir treatment for CMV retinitis, COVID-19 visual impairment risks

## Abstract

Purpose: Cytomegalovirus (CMV) retinitis is a sight-threatening condition predominantly affecting immunocompromised individuals, such as those with Human Immunodeficiency Virus (HIV)/Acquired Immunodeficiency Syndrome (AIDS). We aimed to present an observational case report on CMV retinitis following Severe Acute Respiratory Syndrome Coronavirus 2 (SARS-CoV-2) infection and to review the literature on the molecular and cellular changes in CMV and SARS-CoV-2 infections and how they may influence each other. **Case Description:** A 32-year-old man with a history of AIDS presented with decreased vision and ocular pain exacerbated by movement, beginning a day prior. Ocular examination revealed anterior uveitis, corneal endothelial edema, and retinal necrosis in the left eye. CMV retinitis was diagnosed based on positive serologic testing and a low cluster of differentiation 4 (CD4) count, with concurrent SARS-CoV-2 infection detected. Treatment included valganciclovir and topical agents, with a focus on managing CMV complications. This case highlights the potential role of SARS-CoV-2 in reactivating dormant CMV in severely immunocompromised individuals. We also discuss the implications of this interaction for immunocompromised patients, emphasizing the need for vigilant monitoring and personalized treatment strategies. **Conclusions:** Our case suggests that SARS-CoV-2 may trigger reactivation of CMV infection, leading to bilateral involvement in patients with low CD4 lymphocyte counts, which can result in severe visual impairment. The review discusses the molecular and cellular interactions between CMV and SARS-CoV-2, as well as risk factors, pathophysiology, and diagnostic methods for CMV retinitis, providing recommendations based on the literature findings.

## 1. Introduction

Retinitis caused by cytomegalovirus (CMV) is a sight-threatening condition affecting the retina. Cytomegalovirus is a member of the herpesvirus family and typically remains dormant in the body after the initial infection. However, in individuals with weakened immune systems, such as those with Human Immunodeficiency Virus (HIV)/Acquired Immunodeficiency Syndrome (AIDS) or undergoing immunosuppressive therapy, the virus can clinically reactivate and lead to various complications, including CMV retinitis [1,2].

CMV retinitis primarily targets the retina, causing and leading to various symptoms, such as blurred vision, floaters, and, if left untreated, can progress to severe vision loss or even blindness. The condition often affects individuals with compromised immune systems, making them more susceptible to opportunistic infections [1].

Diagnosis of CMV retinitis typically involves a comprehensive eye examination, including a dilated fundus exam and imaging studies such as optical coherence tomography (OCT) and fluorescein angiography (FA). Early detection is crucial for effective management, as delayed intervention can lead to irreversible vision loss [3,4,5].

Treatment of CMV retinitis usually involves antiviral medications, such as ganciclovir or foscarnet, administered intravenously or through intraocular implants. These medications aim to control the progression of the infection and preserve vision. In some cases, maintenance therapy, such as continued use of antivirals at lower doses or intraocular implants, may be necessary to prevent recurrence [6].

Prevention of CMV retinitis is largely focused on managing the underlying immune-compromising condition, such as HIV/AIDS, through antiretroviral therapy. Regular eye exams and prompt medical attention for any visual symptoms are essential for individuals at risk of CMV retinitis, enabling early detection and intervention to minimize the impact on vision [1].

CMV retinitis has become less common in certain populations, particularly in individuals with HIV/AIDS. The introduction and widespread use of highly active antiretroviral therapy (HAART) have been a major contributing factor to the decline in the incidence of CMV retinitis. HAART has significantly improved the immune function of individuals living with HIV/AIDS by suppressing the replication of the virus and increasing cluster of differentiation 4 (CD4) T-cell counts. Before starting this therapy, approximately 30% of the patients with AIDS have developed CMV retinitis. Other studies have also shown that retinitis usually occurs at CD4 count less than 50 cells/μL. As a result of HAART therapy, the immune system is better equipped to control opportunistic infections, including CMV. With effective HIV management, the prevalence of severe immunosuppression has decreased in 75% of cases, leading to a decline in the occurrence of CMV retinitis [1,6].

Additionally, routine screening and early detection of HIV, along with improved access to healthcare and antiretroviral medications, have played a crucial role in preventing the progression to advanced stages of HIV/AIDS and associated complications like CMV retinitis. Public health initiatives, increased awareness, and improved treatment strategies have collectively contributed to the reduced incidence of CMV retinitis in certain populations [7].

The seventh member of the human coronavirus family, SARS-CoV-2, possesses pandemic properties, which have caused several adverse impacts on humans [8]. It is still unclear how SARS-CoV-2 and herpesviruses, such as CMV, interact with one another. After contracting SARS-CoV-2, the immune system becomes dysregulated at the cellular and molecular levels as a result of a sequence of hyperinflammatory processes [8].

It is important to note that the prevalence of CMV retinitis may vary across different regions and populations, and ongoing research and healthcare efforts are essential to monitor and address the evolving landscape of infectious diseases, including those related to immunosuppression [2,7]. 

## 2. Case Study

**Figure 1:**
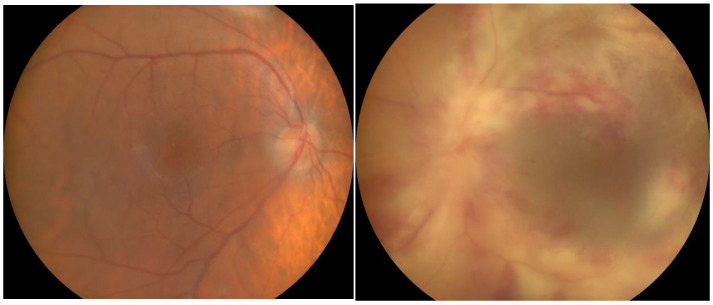
Fundus photographs of the right and left eyes. At presentation, the right eye shows small peripapillary infiltrated zone of white necrosis and the left eye shows pseudopapilledema, with hemorrhagic necrosis on white/yellow cloudy retinal lesions.

A 32-year-old Caucasian man presented at the Ophthalmology Department complaints of decreased vision and ocular pain accentuated during movement that appeared a day before. A week before the onset, the patient also had symptoms specific to a transient ischemic attack, with the loss of muscle force on the right upper limb and aphasia. On ocular examination, his vision in the right eye was 6/6 and in the left eye was HM (hand motion). Slit lamp biomicroscopy, which represent the instrument used to examine the anterior and posterior segments of the eye under high magnification, showed signs of anterior uveitis, with discrete corneal endothelial edema and micro Tyndall, in the right eye and severe corneal endothelial edema—granulomatous inferior inflammatory cells deposited on the cornea-keratic precipitates—and moderate Tyndall in the left eye. Fundus examination showed a small peripapillary infiltrated zone of white necrosis and in the extreme nasal periphery a zone of white necrosis with edema located along the distribution of retinal vessels in the right eye. In the left eye, the fundus examination showed pseudopapilledema, with hemorrhagic necrosis on white/yellow cloudy retinal lesions, centered around vasculature (Figure 1). OCT of the left eye showed necrosis of the fovea, resulting in significant destruction of the retinal nerve fiber layer.

The patient had a diagnosis of AIDS for a few years (since 2017), without being under antiretroviral therapy. We performed laboratory diagnosis of CMV retinitis, which was confirmed with positive results of serologic testing for CMV (IgM and IgG) along with CD4 count of 16 cells/μL, CD8 count of 521 cells/μL, and a ratio of CD4/CD8 lymphocytes of 0.03. Additionally, a COVID-19 reverse transcription-polymerase chain reaction (RT-PCR) test was positive for SARS-CoV-2, though the patient reported only mild symptoms, including a low-grade fever and fatigue, which resolved within a few days without significant respiratory involvement. Interleukin-6 (IL-6) levels were significantly elevated at 180 pg/mL, accompanied by a C-reactive protein (CRP) level of 29.8 milligrams per liter (mg/L).

**Figure 2:**
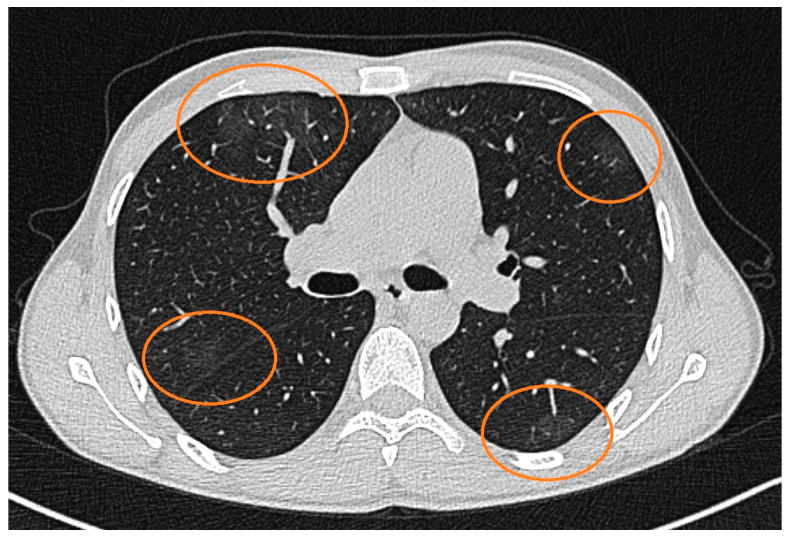
CT scan of the chest showing axial cross-sectional images of the lungs with multiple areas marked in orange circles. These marked areas represent ground-glass opacities. The opacities are distributed in both lungs and subpleural zones, suggesting a diffuse, bilateral pattern. The findings indicate a mild form of SARS-CoV-2 infection, with all lung lobes being affected.

CMV retinitis is considered a stage 4 condition according to the World Health Organization (WHO) staging for AIDS, and can represent clinical failure as well as immunological failure. Induction phase of CMV retinitis was started with tablet valganciclovir 900 mg two times a day (BID) for one month, along with twice weekly monitoring of complete blood count, serum electrolytes, and renal function test. The anterior chamber inflammation was treated using topical mydriatic-agents, hypotensors, and corticosteroids during this stage. Given the mild nature of the COVID-19 infection (Figure 2), no specific antiviral treatment for SARS-CoV-2 was administered, and the focus remained on managing the CMV-related complications. A follow-up SARS-CoV-2 test was performed two weeks later, which remained positive, indicating persistent viral presence. However, by the fourth week, a subsequent RT-PCR test returned negative, confirming that the patient had cleared the SARS-CoV-2 infection after approximately four weeks. 

**Figure 3:**
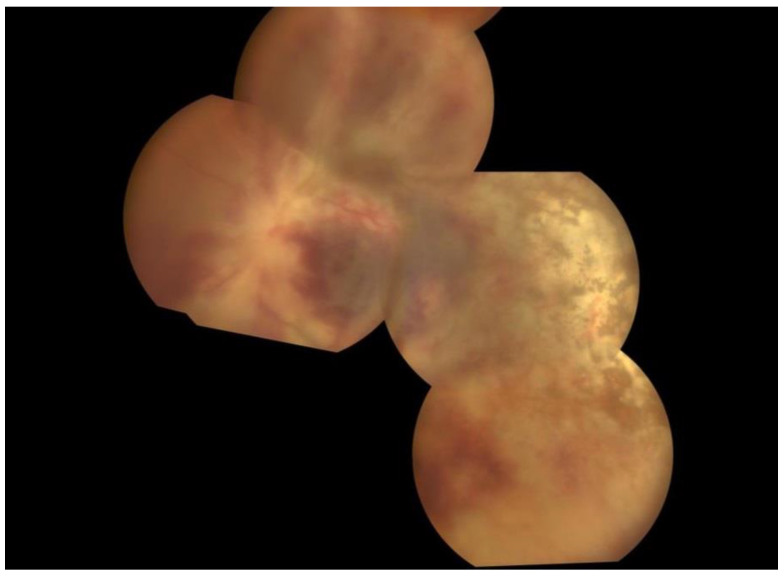
Wide-field fundus photographs of the left eye. One month after the first presentation, the left eye shows moderate remission of active inflammation and hemorrhages.

Eye examination at the end of induction therapy (one-month follow-up) showed the same visual status in the right eye and the worsening of the visual status in the left eye to complete blindness, know as no-light perception (NLP). Anterior chamber in both eyes showed no active inflammation as a sign of anterior uveitis, with no sequelae. Fundoscopy showed dramatic improvement in the retinal white zones (retinal pale areas) in the right eye, with remission of active inflammation. Meanwhile, the left eye showed pale ON (optic nerve), with moderate remission of active inflammation and hemorrhages (Figure 3). 

**Figure 4:**
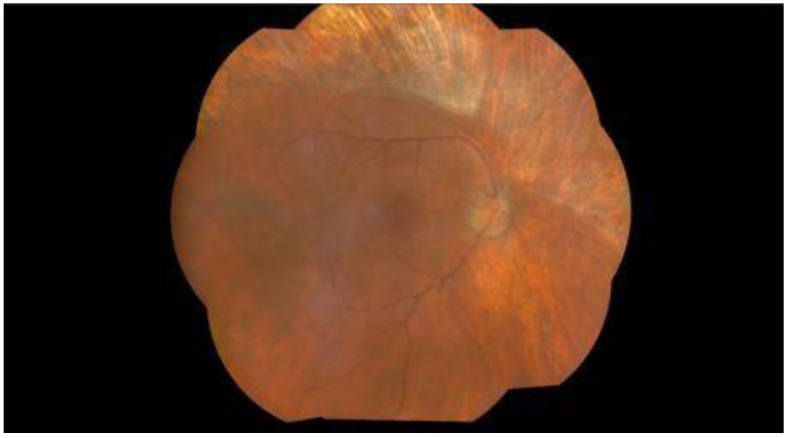
Wide-field fundus photographs of the right eye. Four months after the first presentation, the right eye shows no signs of CMV reactivation or complications.

**Figure 5:**
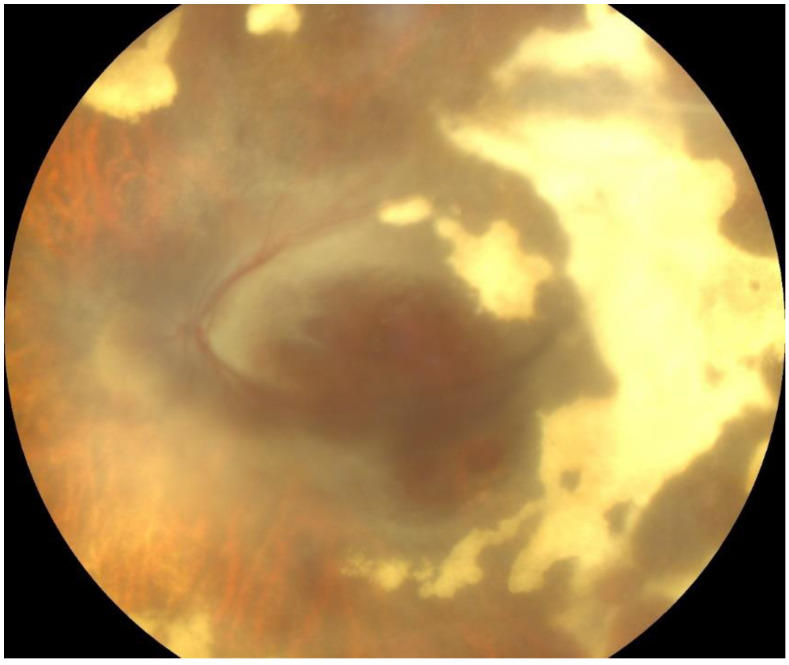
Fundus photographs of the left eye. Four months after the first presentation, the left eye shows scarring at the site of previous retinitis.

Maintenance therapy with 900 milligram (mg) tablets of valganciclovir was continued for 4 months, when fundus examination showed scarring at the site of the previous retinitis in the left eye but no improvement in the visual status (Figure 4 and Figure 5). CMV retinitis has also led to a very serious complication of retinal detachment in his left eye. His follow-up during the next months showed no recurrence of CMV retinitis lesions or evidence of any disseminated CMV disease.

Given the patient’s history and the timing of the SARS-CoV-2 infection, it is plausible that the COVID-19 infection triggered the reactivation of CMV retinitis, which had been dormant due to the severe immunosuppression from untreated AIDS. The markedly elevated IL-6 levels at 180 mg/mL during the acute phase, along with increased CRP levels at 29.8 mg/L, supported the presence of a robust inflammatory response, likely exacerbating the retinitis. This case highlights the potential for SARS-CoV-2 to act as a trigger for CMV reactivation in severely immunocompromised individuals, emphasizing the need for vigilant monitoring and prompt intervention in such cases.

## 3. Discussion

CMV retinitis is commonly identified in individuals with compromised immune systems, particularly those with HIV. In a study conducted by Visser [1], it was observed that around 30% of AIDS patients developed CMV retinitis before initiating HAART. Following the start of HAART, the incidence rate decreased in 75% of cases [9]. Nonetheless, there have also been reports of patients developing CMV retinitis while receiving HAART [10].

### 3.1. Pathogenesis of CMV Retinitis

With a double-stranded deoxyribonucleic acid (DNA) length of 235 kilobase pairs (kbp), CMV is the largest known Herpesviridae virus. An icosahedral protein capsid encased in a lipid bilayer contains the viral DNA. Virion formation and replication are facilitated by the protein capsid and lipid bilayer after invasion of the host [11]. Patients with CMV may not show positive blood tests for viral DNA, although the virus has remained latent in the bone marrow and blood [12]. Reactivation of the virus happens when the host immune system is seriously weakened [13]. Leukocyte depletion during blood transfusion significantly decreased the spread of CMV, according to studies, which prompted additional investigation and discovered that peripheral blood monocytes contained the latent CMV genome [14,15]. It has been demonstrated that cells of the myeloid lineage-cluster of differentiation (CD)34^+^ precursor cells, along with circulating CD14^+^ monocytes derivates, are the reason for CMV latency [16,17,18]. Moreover, CMV reactivation and the release of the infectious virus from its latent location depend on CD34^+^ maturation into dendritic cells [19,20]. The findings of Poole et al. [18] that in vivo–differentiated macrophages assist HCMV reactivation also reinforce experimental latency models, helping to uncover key mechanisms of HCMV latency and reactivation in myeloid cells. Similar to several viruses that avoid host cell immune responses in order to survive, CMV possesses an interleukin-10 (IL-10) homolog that aids in preventing the recruitment of inflammatory and natural killer cells [21].

Ocular active CMV mostly infects vascular endothelial cells, which are then followed by retinal pigment epithelial cells, producing afterwards a viral cytopathic impact and ensuing retinal necrosis. CMV’s massive viral genome rapidly penetrates the nucleus of the infected cell and initiates the lytic lifecycle, which produces viral progeny [22,23]. From the infected cell, the viral progeny branch out to infect nearby cells and continue the process. Unregulated virus multiplication causes cell death in the affected retinal tissue, which eventually results in visual impairment [24]. Chien et al. discovered that in a mouse model of CMV retinitis, the evolution of the pathology involves several mechanisms of cellular destruction, including apoptosis, necroptosis, and pyroptosis. They also discovered that viral infection was not the only cause of cellular death and the clinical symptoms associated with CMV retinitis [25].

### 3.2. Risk Factors Associated with CMV Retinitis

#### 3.2.1. HIV/AIDS

HIV remains a major global health issue with severe health, social, and economic impacts, particularly due to its ability to persist in latent reservoirs. AIDS, defined by a CD4^+^ count below 200 cells/mm^3^ or the presence of an AIDS-defining illness, leads to severe immunosuppression and life-threatening complications [26,27,28]. CMV retinitis is the most common ocular opportunistic infection in AIDS patients, responsible for the majority of CMV-related illnesses and nearly 90% of HIV-related blindness. It typically occurs in the late stages of HIV when CD4^+^ counts fall below 50 cells/μL. While HAART has improved survival and visual outcomes, CMV retinitis still progresses in developing countries where access to treatment is limited [29]. The most critical predictor for CMV retinitis is a CD4^+^ count below 50 cells/µL, with other risk factors including male gender, high HIV viral load, low CD8^+^ T-cell counts, and lack of HAART therapy. Although HIV viral load may influence CMV reactivation, its role is less significant than that of CD4^+^ counts, with conflicting evidence in the literature [30,31,32,33].

#### 3.2.2. Organ and Hematopoietic Stem Cell Transplantation

The diagnosis and treatment of CMV retinitis following hematopoietic stem cell transplantation (HSCT) have gained more attention recently, but data on recurrence after treatment are limited. Two key factors in CMV retinitis development are CD4^+^ T lymphocyte count and CMV viral load in the aqueous humor [34]. Research shows a significant decrease in T lymphocyte counts six months post-HSCT in CMV retinitis patients, with a higher incidence in recipients from mismatched donors due to their weaker immune response [35,36,37,38]. Wang et al. noted that patients with recurrent CMV retinitis post-HSCT had higher CD4^+^ T-cell counts than previously reported [39]. They highlighted that even moderate immunosuppression can lead to clinical CMV reactivation, and ophthalmoscopy alone is insufficient for detecting recurrence. Recurrent CMV retinitis often presents with acute inflammatory reactions, and studies indicate that active retinal CMV infection can persist despite negative CMV antigenemia [40,41,42].

CMV retinitis is also a rare but serious complication in solid organ transplant recipients, increasing the risk of complications, graft loss, and mortality. It has been observed after heart, kidney, and liver transplants, though preventive strategies have altered its epidemiology [43,44,45,46].

#### 3.2.3. SARS-CoV-2 Infection

The mortality associated with COVID-19 is importantly influenced by several known risk factors such as age, ischemia, arterial hypertension, diabetes mellitus, and primary and secondary immunosuppression and can lead to many manifestations of the disease [8,47,48,49,50]. As CMV reactivation in severely immunocompromised patients has been linked with higher morbidity and mortality, it is important to maintain a high suspicion of disease reactivation. Although ocular CMV clinical reactivation following COVID-19 has yet to be widely discussed, several research articles regarding human CMV clinical reactivation have been published in the last few years [51,52]. Krishna et al. have acknowledged the possibility of a reactivation of CMV as well as other latent viruses due to COVID-19, postulating that lymphopenia and inflammation associated with this disease could lead to reactivation [53]. The findings of Perera et al. demonstrate that in human umbilical vein endothelial cells, CMV dramatically upregulates angiotensin converting enzyme 2 (ACE2). Because of their involvement in thrombosis and cytokine production, which both contribute to severe COVID-19 symptoms including clotting and inflammation, endothelial cells are essential in SARS-CoV-2 pathology [52]. Although endothelial cells are not easily infected by SARS-CoV-2 and usually express little to no ACE2, according to previous research [54], their data indicate that CMV upregulates ACE2, maybe as a side consequence of released cytokines, such as IL-6. Their in vitro experiments have shown that this mechanism could boost susceptibility to SARS-CoV-2 infection [52]. 

One study excludes SARS-CoV-2 as an independent risk factor for the reactivation of CMV and suggests that the reactivation is due to the critical condition, rather than the virus [55]. Moreover, high-dose corticosteroid therapy required in such cases may lead to the reactivation [56,57]. 

There are a few articles in the specialized literature studying the post-COVID-19 reactivation of CMV infection. In critically ill COVID-19 patients, there is a notable higher occurrence of CMV blood reactivation (20.4% of patients) [32]. The risk of CMV reactivation is closely tied to the severity of the illness and the onset of secondary bacterial infections [58]. Ongoing research is needed to elucidate the specific role of CMV reactivation in the context of severe COVID-19 and to establish effective strategies for its appropriate management.

The complex interplay between these viral infections can exacerbate the severity of the patient’s condition, with each virus potentially influencing the course of the other. This bidirectional interaction means that once one virus is active, it can trigger or worsen the effects of the other, leading to a compounded immunological response and increased disease severity.

Patients with acute or reactivated immune system dysfunction-related disorders, such CMV retinitis, may need to be evaluated for a potential SARS-CoV-2 infection [59]. Furthermore, in the event of a SARS-CoV-2 infection, patients with a personal history of illnesses like CMV disease, where they may be susceptible to immune system dysregulation, should be examined for possible reactivations. The hazards of long-term antiviral medication may be exceeded by danger of potential visual loss in the contralateral eye. Patients having a history of CMV retinitis or any other ocular HIV-related ocular manifestation may be more susceptible to disease reactivation due to the altered immunological state caused by COVID-19. As such, they should think about secondary antiviral treatment during SARS-CoV-2 infection. There is no specific antiviral drug that targets both SARS-CoV-2 and CMV simultaneously. In clinical practice, CMV reactivation during COVID-19 may necessitate the introduction of standard CMV antivirals alongside COVID-19 therapies. Timely management with ganciclovir is associated with favorable outcomes in CMV infection; one study suggested that ganciclovir treatment should be initiated in COVID-19 patients with high CMV viral loads [60]. Limaye et al. observed no variations in ganciclovir treatment versus placebo groups’ IL-6 levels, length of mechanical ventilation, or mortality; however, ganciclovir prophylaxis was linked to a reduced rate of CMV reactivation [61]. In a trial conducted by Cowley et al., critically ill patients receiving care in the intensive care unit for various illnesses were randomized to receive valacyclovir, valganciclovir, or a placebo. The results showed that although prophylaxis with any antiviral drug was linked to a lower incidence of CMV reactivation when compared to a placebo, valacyclovir prophylaxis was linked to a higher mortality rate when compared to both alternatives [62]. 

Studies have reported that vaccination against COVID-19 might lead to reactivation of dormant viruses [63,64], which can pose a threat to vision and the quality of life. However, these studies have only reported this complication, without confirming it. 

#### 3.2.4. Medication-Induced

CMV reactivation, though rare, has been reported in HIV-negative patients on chronic corticosteroid therapy for non-infectious uveitis, such as Behçet uveitis and Vogt–Koyanagi–Harada disease (VKH) disease [65,66,67]. CMV retinitis has also been seen in patients with normal CD4 counts on corticosteroids or immunosuppressants like azathioprine for other diseases [68].

## 4. Pathophysiological and Immunological Viral Interactions

CMV is a widespread herpesvirus that leads to prolonged infection, impacting both the immune system’s innate and adaptive components. While it has been implicated in immunosenescence, the exact role of CMV in this process remains debated, as multiple mechanisms are associated with aging and immune decline [69]. Chronic CMV infection, which involves a range between 40% and 90% of the world’s population, has lately been related to autoimmune and cardiovascular disease, with prevalence varied by age, wealth, race, ethnicity, and education. CMV reactivation has emerged as a potential risk factor for severe COVID-19, particularly in immunocompromised individuals or those with underlying health conditions. The immune dysregulation caused by SARS-CoV-2, characterized by lymphopenia, cytokine storm, and reduced T-cell function, creates an environment conducive to CMV reactivation. This reactivation can exacerbate the inflammatory response, contributing to organ damage and worsening the severity of COVID-19 [59,70]. The study of Kew et al. [70] indicates that inflammation may lead to CMV reactivation by causing or enhancing the production of the immediate early (IE) genes from a previously quiescent state. Additionally, CMV reactivation may impair the body’s ability to mount an effective immune response against SARS-CoV-2, increasing the risk of complications. Moss et al. have proposed that CMV may trigger metabolic and cardiac issues in COVID-19 patients [52]. Severe inflammation may reactivate CMV in approximately four to seven days, which is equivalent to in vitro reactivation. As a result, SARS-CoV-2’s strong immune system stimulation may cause CMV reactivation in organs, such as the lungs and colon [71].

Several genes within the CMV genome can evade the host immune system, with viral interleukin-10 (IL-10) being one of the most studied [7,72,73]. Viral IL-10 suppresses the production of pro-inflammatory cytokines, downregulates the expression of major histocompatibility complex (MHC) class II, and reduces the expression of co-stimulatory molecules, thereby inhibiting inflammatory and cell-mediated immune responses [74]. The IL-10/IL-10R signaling pathway is crucial for viral persistence, and findings by Sezgin et al. suggest that blocking the IL-10/IL-10R interaction could be beneficial in controlling chronic CMV infections [75]. The chemokine receptor 5, also known as CCR5, is a protein thought to play a key role in HIV cell entry. A specific promoter haplotype of CCR5, known as the + .P1. + haplotype, has been associated with faster progression to AIDS and delayed HIV viral suppression, particularly in African-American individuals. This haplotype is linked to rapid disease progression in the early years after infection. Additionally, the CCR5 + .P1. + haplotype negatively affects viral suppression and CD4^+^ T-cell response to HAART treatment [76].

Both CMV and SARS-CoV-2 infections not only upregulate cyclooxygenase-2 (COX-2) but also lead to elevated levels of IL-6, a key pro-inflammatory cytokine. In CMV infection, IL-6 is produced as part of the host’s immune response, contributing to the persistence and reactivation of the virus. The virus is thought to manipulate host signaling pathways to increase IL-6 production, thereby enhancing inflammation and promoting an environment conducive to viral replication and immune evasion [71,77]. However, comprehending the molecular and cellular underpinnings of reactivation of HCMV is still an elusive issue. Similarly, SARS-CoV-2 infection triggers an important IL-6 response, which plays a central role in the cytokine storm associated with severe COVID-19. Elevated IL-6 levels drive systemic inflammation, leading to acute respiratory distress syndrome (ARDS), coagulopathies, and multi-organ failure. The mechanisms behind IL-6 upregulation in SARS-CoV-2 involve the activation of various signaling pathways, including the nuclear factor kappa B (NF-κB) and signal transducer and activator of transcription 3 (STAT3) pathways, which amplify the inflammatory response and contribute to the severe clinical outcomes observed in COVID-19 [77].

In both CMV and SARS-CoV-2 infections, the upregulation of COX-2 and IL-6 creates a feedback loop that exacerbates inflammation. COX-2 increases prostaglandin production, which further stimulates IL-6 release, while IL-6 can enhance COX-2 expression, perpetuating the inflammatory cycle. This interaction between COX-2 and IL-6 shows the pathophysiological similarities in how these viruses manipulate the host immune response, leading to severe inflammatory conditions and complications. The CMV protein US28 modulates COX-2 activity and phosphorylates STAT3, which leads to the generation of vascular endothelial growth factor (VEGF) and IL-6 and causes smooth muscle cell proliferation connected to cardiovascular disorders [78]. IL-10’s immunosuppressive properties can also be exploited by CMV to prolong its persistence in the host by inhibiting the activity of cytotoxic T cells and other immune effector functions [79]. In SARS-CoV-2 infection, IL-10 is similarly upregulated as part of the body’s attempt to control inflammation. However, in the context of a cytokine storm, the simultaneous elevation of IL-10 and pro-inflammatory cytokines reflects a dysregulated immune response [80]. TGF-β facilitates immune tolerance and can contribute to the establishment of chronic CMV infection by inhibiting the activation and proliferation of T cells. Similarly, in SARS-CoV-2 infection, TGF-β is often elevated and may contribute to the fibrotic changes observed in the lungs of patients with severe COVID-19 [81]. 

In the context of CMV, macrophages undergo significant changes that can further influence the inflammatory landscape. CMV can alter macrophage function by enhancing their pro-inflammatory activity and altering cytokine production, including increasing IL-6 and TNF-α levels. This can intensify the inflammatory response in COVID-19 patients, particularly those with reactivated CMV [82]. CMV-infected macrophages may also contribute to immune dysregulation, compounding the effects of SARS-CoV-2 by further disrupting normal immune responses and exacerbating the cytokine storm. CMV-infested epithelial cells may produce monocyte chemotactic protein 1 (MCP-1), macrophage inflammatory protein (MIP)-1α, and interferon-gamma-induced protein 10 (IP10), leading to widespread pulmonary macrophage infiltration. Many of the invading macrophages contain latent CMV that can be reactivated by an inflammatory reaction, which is a common hallmark of COVID-19. SARS-CoV-2 infection stimulates macrophages by initiating an ongoing process of M1-type macrophage polarization, which promotes CMV reactivation and fuels additional inflammatory response [83]. The combined impact of SARS-CoV-2 and CMV on macrophages and cytokine production highlights the relationship between these viruses and the host’s immune system, contributing to the severity of COVID-19. TNF-α is rapidly upregulated in response to these viral infections, primarily through the activation of the NF-κB signaling pathway [83].

**Figure 6:**
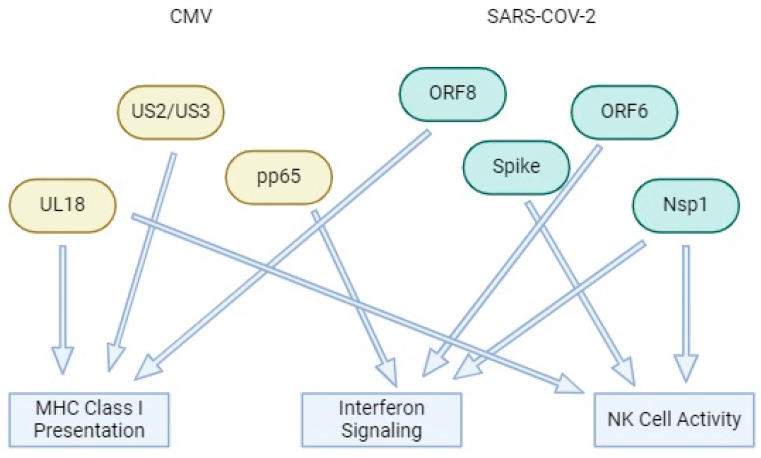
Viral protein interactions in CMV and SARS-CoV-2 infections: mechanisms of immune suppression. Abbreviations: UL18—unique long 18; pp65—phosphoprotein 65; US2/US3—unique short 2/3; ORF6/ORF8—open reading frame 6/8; Nsp1—non-structural protein 1; MHC-I—major histocompatibility complex class I; NK cells—natural killer cells; IFN—interferon.

Both CMV and SARS-CoV-2 utilize specific viral proteins to evade the host immune system, leading to immune suppression. CMV proteins, such as unique lone (UL)18, mimic MHC Class I molecules to evade T-cell detection [84], while phosphoprotein 65 (pp65) interferes with IFN signaling, reducing the antiviral response [85]. Proteins unique short (US)2 and US3 further block MHC Class I presentation, preventing the display of viral peptides that would normally alert cytotoxic T cells [86]. In parallel, SARS-CoV-2 proteins like Spike affect natural killer (NK) cell activity, helping the virus evade immune surveillance [87], while both ORF6 and Nsp1 of SARS-CoV-2 suppress IFN signaling [88]. Together, these viral proteins from both CMV and SARS-CoV-2 target overlapping immune pathways (Figure 6), such as MHC-I presentation and IFN signaling, resulting in enhanced viral survival and propagation in the host by dampening both innate and adaptive immune responses [87,88]. 

## 5. Biomarkers

The discovery of new biomarkers is essential to efficient management and medical treatment [89]. The investigation of Kato et al. examined a profile of biomarkers in connection to different COVID-19 phenotypes. Comparing severe cases to moderate, asymptomatic, and healthy subjects, they discovered that the plasma levels of IFN-γ, TNF-α, IL-6, IL-10, ferritin, homocysteine, and D-dimers were significantly higher in the former cases [90]. The systemic inflammatory responses linked to these increased inflammatory biomarkers exacerbate the disease and worsen the prognosis. Significantly elevated D-dimers and ferritin levels were observed; D-dimer levels above 1 µg/mL were linked to a poor prognosis, and values ≥ 2 µg/mL were predictive of mortality [91,92]. Elevated ferritin has been suggested as a prognostic biomarker for the advancement of this disease [93]. Comparably, it has been demonstrated that C-reactive protein (CRP), which has a good diagnostic accuracy in identifying severe COVID-19, correlates with the severity of the disease and was markedly elevated in severe instances [94]. Severe disease was also linked to elevated homocysteine and Angiotensin II levels, with Angiotensin II playing a role in the cytokine storm and unfavorable outcomes. Consistent with previous research, upregulated cytokines signify immunological activation and are associated with a poor prognosis and severity of the disease [95,96]. These biomarkers may be helpful in controlling treatment, forecasting the severity of the disease, and evaluating the results in COVID-19 patients.

One of the primary immunological markers involved in CMV reactivation is the CMV-specific T-cell response. CD8^+^ cytotoxic T lymphocytes (CTLs) are particularly important in controlling CMV replication. A decrease in the frequency or functionality of CMV-specific CTLs has been associated with an increased risk of CMV reactivation [97]. Other important inflammatory and immunological markers in CMV reactivation are sCD14, IL-6, sCD163, and IP10. Temporary inhibition of the infection may be achieved by a rise in CMV IgG and CMV-specific T lymphocytes, but further cycles of inflammation and reactivation are likely to cause harm to cells and tissues [98]. Furthermore, the monitoring of CMV-specific immunoglobulin G (IgG) and immunoglobulin M (IgM) antibodies can also be useful. IgG antibodies indicate past infection and immunity, while a rise in IgM suggests recent or active reactivation [99,100]. CD163^+^ macrophages, CD68^+^ macrophages, and CD3^+^ T-cell infiltrates are located in juxtaposition to CMV-positive cells during persistent ocular CMV infection [101]. Furthermore, elevated expression of IL-5, IL-6, IL-8, chemokine ligand (CCL)-2, CCL-4, granulocyte-colony stimulating factor (G-CSF), and TGF-β have been found in the aqueous humor of CMV-infected patients [101]. 

Recent advancements have also focused on the role of immune checkpoint molecules, such as programmed cell death protein 1 (PD-1), which can modulate T-cell exhaustion during CMV reactivation. Increased expression of PD-1 on CMV-specific T cells has been linked to poor control of viral replication, making it a potential marker for CMV reactivation risk [102].

## 6. CMV Reactivation as a Novel Risk Factor for Severe COVID-19

CMV has emerged as a potential novel risk factor for increased severity in COVID-19, particularly among immunocompromised individuals. CMV may reactivate during periods of immune suppression, leading to a range of complications. Retrospective studies have attempted to find evidence of CMV reactivation during severe COVID-19 [58,81], but have so far been inconclusive. The challenge lies in the complexity of detecting CMV reactivation, which requires optimal sample collection and precise diagnostics. Consequently, new prospective studies are being initiated to specifically investigate CMV reactivation in severe COVID-19 cases.

The study of Pothast et al. found that CMV-specific T cells cross-react with SARS-CoV-2, even if they share low sequence homology, and might contribute to the pre-existing immunity against SARS-CoV-2 [103]. The discovery of CMV/SARS-CoV-2 cross-reactive T cells suggests that CMV infection may represent a contributing factor in severe COVID-19 by selective activation of T cells from the antigen-experienced or memory T-cell pool. These cells are frequently not as responsive to the antigen for which they had not been initially primed, and as a result, a weakened T-cell response may fail to control SARS-CoV-2, resulting in severe COVID-19.

However, this cross-reactive potential is not exclusive to CMV. Similar cross-reactivity has been observed with T cells specific to other pathogens, such as common cold coronaviruses [104]. These findings imply that individuals may frequently have pre-existing memory against recently encountered antigens and that the proportion of memory versus naive T cells varies with the size of the overall CD4^+^ memory compartment [105]. In severe COVID-19 patients, pre-existing T-cell immunity from prior exposure to these other coronaviruses may contribute to the recruitment of SARS-CoV-2-specific T cells. This cross-reactivity suggests that the immune system’s prior encounters with various pathogens, including CMV and common coronaviruses, could influence the body’s response to SARS-CoV-2, potentially affecting the severity and course of COVID-19 [105,106]. Memory T-cell adoptive therapy represents a promising strategy for treating COVID-19, particularly in patients with compromised immune responses. By harnessing the immune memory of individuals who have recovered from the infection, this therapy has the potential to significantly enhance the host’s ability to combat SARS-CoV-2, thereby mitigating disease severity. However, to fully unlock its therapeutic potential and address associated challenges, further research and extensive clinical trials are imperative. Several researchers are looking into the involvement of SARS-CoV-2-specific T lymphocytes acquired from cross-reactive, antigen-experienced repertoires in COVID-19 [105,106,107]. CMV may be a major component because of its major effect on T-cell repertoire alterations. Considering the proof suggesting that CMV causes immunological decline, particularly among older people, the investigation of Weber et al. [107] discovered a striking link between CMV antibodies and COVID-19 in younger patients. If CMV serology does affect the quality of SARS-CoV-2-specific T-cell responses in extreme cases, adoptive T-cell treatment using extremely specific SARS-CoV-2 T cells may be an attractive treatment option.

## 7. Diagnosis

The diagnosis of CMV retinitis is mostly clinical and is based the patient’s immunological status and the observation of characteristic retinitis on dilated ophthalmoscopic examination. PCR examination of ocular fluids, such as aqueous or vitreous humor, serves as an additional confirmation method for diagnosing cytomegalovirus retinitis. This molecular analysis can be particularly beneficial when clinical presentations are atypical or when confirmation is needed.

### 7.1. Physical Examination

CMV retinitis is suspected when ophthalmic examination reveals a white, necrotic retinal lesion, often with associated hemorrhage, particularly in immunosuppressed adults. Monitoring the lesion’s growth is essential and can be done through regular eye examinations and fundus photography. Growth is indicated either by direct observation of lesion enlargement or by detecting an active lesion adjacent to an atrophic retinal area. The severity and potential threat to vision are assessed by classifying the retinal zones 1–3 [3].

### 7.2. Fluorescein Angiography and OCT

Fluorescein angiography is a key diagnostic tool for assessing CMV retinitis, offering detailed insights into retinal blood circulation and vascular abnormalities. It is particularly effective in revealing hypofluorescent areas during early and intermediate phases, which indicate retinal necrosis and ischemia due to CMV infection. The technique also identifies vasculitis and abnormal fluorescein leakage, signaling active infection and disease progression [4,5].

Ophthalmologists analyze these images to assess the size, location, and extent of lesions, as well as abnormal vasculature, aiding in the diagnosis, severity assessment, and treatment planning for ocular diseases [108]. OCT complements this by monitoring macula-involving CMV retinitis, although the specific microstructural features of active CMV retinitis remain under-researched. Future studies may explore the clinical relevance of OCT findings, such as vitreous inflammation, retinal disruption, and hyperreflective foci [109].

### 7.3. Polymerase Chain Reaction

PCR for the diagnosis of CMV retinitis is a molecular biology technique used to detect the presence of CMV DNA in ocular fluids, such as aqueous or vitreous humor. PCR amplifies specific DNA sequences, allowing for the identification and confirmation of the virus. A positive PCR result indicates the presence of CMV DNA in the ocular fluid, supporting the diagnosis of CMV retinitis. A negative result does not rule out the possibility of the infection, as the timing of sample collection and the distribution of the virus within the eye can influence the test sensitivity.

Several PCR methods can be used for the diagnosis of CMV retinitis, providing sensitive and specific markers that can differentiate active and inactive CMV infection [110]. The choice of PCR method depends on various factors such as sensitivity, specificity, turnaround time, and the ability to detect different CMV strains. Common PCR methods used for the diagnosis of CMV retinitis include conventional PCR, real-time PCR (qPCR), nested PCR, reverse transcription PCR (RT-PCR), multiplex PCR, digital PCR, and in situ PCR [111,112,113,114,115,116]. 

Improving PCR sensitivity for detecting CMV is particularly important in cases involving immunocompromised patients or co-infections. Optimizing primer and probe design is essential, ensuring the selected primers target highly conserved regions of the CMV genome. This increases the accuracy of viral DNA detection, even at low viral loads. Multiplex PCR primers that can amplify multiple regions of the genome offer an additional layer of sensitivity, as they increase the likelihood of capturing viral DNA, particularly in samples where the viral load is low or fragmented [117]. Real-time quantitative PCR (qPCR) is another advancement that can significantly increase sensitivity [118]. For even higher sensitivity, droplet digital PCR (ddPCR) can be employed, which partitions the PCR reaction into thousands of individual droplets. Each droplet contains a small portion of the sample, allowing for highly sensitive detection of low-abundance viral DNA [119]. Addressing PCR inhibitors is another critical aspect of improving sensitivity. Inhibitors from clinical samples, such as blood or tissue, can interfere with the PCR reaction. Using PCR inhibitor removal kits or optimizing extraction methods to eliminate these substances can enhance the sensitivity of the assay [120]. Additionally, incorporating PCR enhancers like BSA or DMSO can help counteract the effects of inhibitors, ensuring more efficient amplification of viral DNA. Optimizing PCR reaction conditions is another key strategy for improving sensitivity. Adjusting parameters such as annealing temperature, magnesium ion concentration, and the number of PCR cycles can fine-tune the reaction to maximize the amplification of CMV DNA [117,118,119]. Careful optimization of these variables is crucial, particularly when dealing with low viral loads. Furthermore, nested PCR, a two-step process where a second round of amplification is performed using primers that target a sequence within the initial PCR product, can provide even greater sensitivity, though it requires more time [118].

### 7.4. CMV Antibodies

While the detection of cytomegalovirus antibodies can play a role in the diagnosis of CMV infection, it may not be the primary method for diagnosing CMV retinitis [121]. CMV antibodies are produced by the immune system in response to the virus, and their presence can indicate a past or present CMV infection. A few disadvantages include seroprevalence (detection of antibodies only indicates exposure, not necessarily an ongoing or recent CMV infection), impaired response in immunosuppressed patients, and limited sensitivity. Serological testing for CMV antibodies can still be valuable in certain situations, such as determining a person’s immune status or assessing the risk of CMV infection in specific populations. It can help identify individuals who are at risk of CMV reactivation or reinfection [122].

### 7.5. Antigen Testing

The gold standard technique for tracking HCMV reactivation has long been the measurement of pp65 antigenemia [123], but it necessitates extremely expert sample processing and interpretation [124]. However, the therapeutic management of this patients has significantly improved since the identification of pp65 antigen, a hallmark of active viral replication. As a result, pre-emptive medication may be a better option than universal prophylactic in lowering the risk of CMV illness. However, the pp65 antigenemia assay has a number of drawbacks that limit its application in clinical settings: it is a manual test, blood samples must be processed within 7–8 h of collection, and reading the slides requires a high degree of technical proficiency. Another important drawback of this test is leukopenia, particularly in patients undergoing hematopoietic stem cell transplant [125,126]. Table 1 presents comparison of diagnostic methods for CMV infection.

## 8. Personalized Treatment

Recognizing CMV as a risk factor for severe COVID-19 could lead to more targeted therapeutic strategies, particularly in the rare cases where CMV reactivation complicates the course of COVID-19. Administering CMV-specific antiviral treatments such as ganciclovir, valganciclovir, or foscarnet could help control CMV replication, potentially reducing the inflammatory burden and mitigating the cytokine storm associated with severe COVID-19. These antiviral therapies might also prevent immune system diversion towards CMV, allowing a more effective response against SARS-CoV-2, thereby reducing the risk of complications like acute respiratory distress syndrome (ARDS) and multi-organ failure [59,81,85]. Dexamethasone has been widely recognized as an effective anti-inflammatory treatment for COVID-19, particularly in patients with severe respiratory symptoms. Severe respiratory disease in COVID-19 is often driven by an exaggerated immune response, leading to acute respiratory distress syndrome (ARDS) and lung injury [127,128]. Clinical trials have shown that dexamethasone significantly reduces mortality in hospitalized patients requiring oxygen or mechanical ventilation by dampening the excessive inflammatory response associated with severe COVID-19 [129].

In cases where CMV retinitis co-occurs with COVID-19, intraocular antiviral therapies, such as intravitreal injections of ganciclovir or foscarnet, should be administered only after a confirmed diagnosis of CMV retinitis, and not as a prophylactic measure, alongside systemic treatment [33,35]. Letermovir could also be a treatment option in drug-resistant CMV retinitis; however, clinicians should be aware of treatment failure of emergence of resistance in these cases [130]. These approaches aim to preserve visual function by controlling retinal viral load while simultaneously managing the systemic effects of both CMV and SARS-CoV-2. Monitoring retinal health in COVID-19 patients with known CMV seropositivity or reactivation is crucial for early detection and treatment of CMV retinitis, which can lead to significant vision loss if untreated. Novel treatments for CMV retinitis are being studied, including a randomized, placebo-controlled, multicenter trial (MACRT) that was conducted to evaluate the efficacy and safety of a monoclonal anti-CMV antibody treatment (MSL-109) as adjunct therapy to ganciclovir or foscarnet for prolonging the time to relapse in patients with CMV retinitis, including both newly diagnosed and relapsed cases (NCT00000135, NCT00000836, NCT00002016). Clinical trials provide crucial data on safety, efficacy, and optimal usage, which can lead to the development of innovative drugs and treatment protocols [131]. Another study is comparing the effectiveness of combined foscarnet and ganciclovir therapy with cidofovir for treating relapsing CMV retinitis (NCT00000894). This trial aims to identify the most effective regimen for managing relapses, potentially improving outcomes for patients with recurrent infections. Another clinical trial aims to determine if co-administration of sargramostim (GM-CSF) improves ganciclovir tolerance in patients with neutropenia, and whether this improved tolerance leads to delayed progression of CMV retinitis. Additionally, it evaluates the safety of the combined therapy, and assesses changes in HIV p24 antigen expression and T4^+^ lymphocyte counts in patients receiving ganciclovir with or without GM-CSF (NCT00002070, NCT00000989). Researchers are also trying to determine the maximum tolerated dose (MTD) and dose-limiting toxicities of a regimen combining therapeutic ganciclovir, antiretroviral therapy, and recombinant interleukin-2 (Proleukin) in HIV-positive patients. It also investigates the effect of increasing doses of Proleukin on the progression time of CMV retinitis and evaluates the incidence and level of anti-interleukin-2 (anti-IL-2) antibody formation in patients receiving subcutaneous Proleukin (NCT00002321).

The potential interaction between CMV antivirals, such as ganciclovir or valganciclovir, and COVID-19 antivirals, such as Paxlovid (nirmatrelvir/ritonavir), is an important consideration for clinicians managing patients with co-infections. Paxlovid includes ritonavir, a strong CYP3A4 inhibitor, which can interact with other medications that are metabolized by the CYP enzyme system [132], including certain antivirals used for CMV. Ganciclovir and valganciclovir, while not primarily metabolized by CYP enzymes, could potentially interact with medications that affect renal function, given that both drugs are renally excreted [133]. Given the potential for nephrotoxicity and hematologic toxicity, patients receiving both CMV and COVID-19 antivirals should be closely monitored for renal function and blood counts.

Vaccination status is a crucial determinant of COVID-19 mortality risk. Evidence shows that individuals who are fully vaccinated, especially those with booster doses, have a significantly reduced risk of severe outcomes and mortality from COVID-19 compared to unvaccinated individuals [134]. Other important factors, such as comorbidities, age, immune status, and socioeconomic factors [135], should also be considered when evaluating mortality risks and treatment in patients with concurrent COVID-19 and CMV infections.

These clinical scenarios, while rare, underscore the need for a nuanced approach to treatment in high-risk groups, particularly those with a history of CMV-related complications [105,106]. From a vaccination policy perspective, understanding the interplay between CMV and SARS-CoV-2 could influence prioritization for COVID-19 vaccination in individuals at risk of CMV reactivation. Although this is a theoretical risk and not a common occurrence, it is an important consideration, particularly in individuals with a history of CMV-related complications or those who are significantly immunocompromised. Personalized treatment could include booster doses or enhanced monitoring to maintain immunity against SARS-CoV-2, especially in populations vulnerable to both viruses. Further research will be essential to integrate these considerations into public health strategies and clinical protocols, ensuring optimal outcomes for these complex cases.

## 9. Limitations

The primary limitation of this study is the small sample size, as it was based on the clinical course of a single patient. This significantly limits the generalizability of the findings and makes it difficult to draw broader conclusions about the interplay between CMV reactivation and COVID-19 severity. While the insights gained from this case study are valuable, it is challenging to extend these findings to a wider population, given the unique nature of this patient’s medical history and condition. As such, the conclusions drawn from this study must be interpreted with caution. Additionally, the severe immunosuppression associated with AIDS alters the patients’ ability to control viral infections, including CMV, and this may influence the outcomes of both viral activity and treatment responses.

Additionally, there is the potential for researcher bias in interpreting the data, as case studies inherently involve detailed subjective analysis. The lack of a larger sample or control group also prevents us from establishing clear cause-and-effect relationships between CMV reactivation and COVID-19. 

The limited sample size further restricts the statistical power of the study and the ability to make broader generalizations. Moreover, the subjective interpretation of data could influence the findings, particularly given that certain advanced diagnostic investigations that might provide more detailed insights into the patient’s condition were not performed due to case-specific constraints. 

Despite these limitations, the study provides valuable insights and highlights the importance of comprehensive follow-up and further research in larger patient populations. The interaction between SARS-CoV-2 and CMV, particularly in the context of ocular pathology, remains underexplored and warrants additional investigation.

## 10. Conclusions

One of the most dangerous ocular side effects in immunocompromised patients is CMV retinitis, which can cause irreversible vision loss. Early in the course of HIV disease, ART optimization can avoid CMV retinitis; however, because HIV patients now live longer, CMV retinitis remains an issue in modern times. Our case suggests that CMV reactivation may happen following SARS-CoV-2 infection. The interplay between CMV and SARS-CoV-2 may exacerbate immune dysregulation, leading to worsened outcomes in patients with co-infections. The literature has shown us that both infection and vaccination against SARS-CoV-2 might lead to virus reactivation and several complications. Large-scale vaccination campaigns are being carried out worldwide, so post-marketing surveillance systems are necessary to evaluate vaccine safety and detect any adverse events. Given that SARS-CoV-2 vaccinations are scheduled for hundreds of millions of people, a plausible causal relationship with CMV reactivation could lead to a significant number of cases with potentially serious sequelae.

## Figures and Tables

**Table 1:** Comparison of diagnostic methods for CMV infection.

Type	Method	Advantages	Disadvantages
PCR (Polymerase Chain Reaction) [111,115,116]	Molecular biology technique used to amplify a specific segment of DNA for detection.	High sensitivity, allowing for the detection of low levels of CMV DNA. Rapid turnaround time, providing results in a matter of hours. Quantitative PCR can monitor changes in viral load over time.	Requires specialized equipment and trained personnel. Costlier compared to some other methods. Susceptible to contamination, which can lead to false positives.
Serology (Antibody Testing) [121,122]	Testing for antibodies produced by the immune system in response to CMV infection. It detects IgM and IgG antibodies specific to CMV in the patient’s blood serum.	Widely available and relatively inexpensive. Useful for determining past exposure or current infection status. Can differentiate between acute and chronic infections based on antibody levels.	IgM antibodies may persist for months, leading to difficulty in distinguishing recent infection from past exposure. Results may take days to weeks to become positive after infection. Limited usefulness in immunocompromised patients who may not produce antibodies effectively.
Antigen Testing [123,124,125,126]	Involves detecting CMV antigens (proteins) directly in patient samples. Commonly used antigens include pp65 or CMV immediate early antigens.	Rapid detection of CMV antigens in clinical specimens like blood or tissue. Useful for diagnosing active CMV infection, especially in immunocompromised individuals. Can be used in conjunction with other methods for diagnosis.	Less sensitive than PCR, potentially leading to false negatives. May require specialized equipment and trained personnel. Limited availability in some healthcare settings compared to serological tests.

## Data Availability

Data are available on demand.
